# Not Just an Urban Phenomenon: Uninsured Rural Trauma Patients at Increased Risk for Mortality

**DOI:** 10.5811/westjem.2015.7.27351

**Published:** 2015-10-20

**Authors:** Azeemuddin Ahmed, Karisa K. Harland, Bryce Hoffman, Junlin Liao, Kent Choi, Dionne Skeete, Gerene Denning

**Affiliations:** * University of Iowa, Department of Emergency Medicine, Iowa City, Iowa; † University of Iowa, Department of Surgery, Iowa City, Iowa

## Abstract

**Introduction:**

National studies of largely urban populations showed increased risk of traumatic death among uninsured patients, as compared to those insured. No similar studies have been done for major trauma centers serving rural states.

**Methods:**

We performed retrospective analyses using trauma registry records from adult, non-burn patients admitted to a single American College of Surgeons-certified Level 1 trauma center in a rural state (2003–2010, n=13,680) and National Trauma Data Bank (NTDB) registry records (2002–2008, n=380,182). Risk of traumatic death was estimated using multivariable logistic regression analysis.

**Results:**

We found that 9% of trauma center patients and 27% of NTDB patients were uninsured. Overall mortality was similar for both (~4.5%). After controlling for covariates, uninsured trauma center patients were almost five times more likely to die and uninsured NTDB patients were 75% more likely to die than commercially insured patients. The risk of death among Medicaid patients was not significantly different from the commercially insured for either dataset.

**Conclusion:**

Our results suggest that even with an inclusive statewide trauma system and an emergency department that does not triage by payer status, uninsured patients presenting to the trauma center were at increased risk of traumatic death relative to patients with commercial insurance.

## INTRODUCTION

In 2012, approximately 48 million people living in the United States (18% of U.S. citizens) lacked health insurance. 1, 2 Studies of the uninsured have consistently shown that lack of insurance is associated with increased mortality, both when all causes of death were included 3–5 and when chronic health conditions such as cancer 6–12 and heart failure 13–15 were independently examined. Researchers hypothesize that the differences in mortality between uninsured and commercially insured patients may be due to a variety of reasons, including treatment delay, improper triage, under-performance of diagnostic tests and decreased health literacy. 1, 16

With respect to traumatic injury, studies using the National Trauma Data Bank (NTDB) have shown that uninsured Americans have a 1.3- to 3.3-fold higher risk of traumatic death, as compared to patients with commercial or private insurance. 1, 17–25 These prior studies have been limited to intentional injuries, 24, 26, 27 to injuries with greater severity, 21, 22, 28 or to a subset of injury mechanisms such as motor vehicle crashes 29 or pedestrian deaths. 25 In addition, NTDB data represent predominately urban trauma centers; i.e. 80% of NTDB-recorded incidents occurred in metropolitan counties with core populations of 50,000 or more. 17–25 No study to date has examined the risk of trauma-related death as a function of insurance status in a largely non-metropolitan, rural population.

Rural America represents approximately 59 million people, 19.3% of the population. 30 Proportionally, more rural Americans are uninsured (9.9%) than urban Americans (8.5%) and poverty rates are higher in rural areas. 31 Both of these factors put rural citizens at higher risk of poor health.

Despite these risk factors, we hypothesized that uninsured trauma patients presenting to an American College of Surgeons-certified Level 1 trauma center in a state with an inclusive trauma system would not be at increased risk of traumatic death, as compared to insured patients from the same population. This hypothesis was based on the fact that the statewide trauma system triages patients based on mechanism of injury, injury types and available resources rather than by payer status. It was also based on a recent study of trauma-related emergency department (ED) visits suggesting that rural settings were more likely to appropriately triage a patient than urban settings. 16 To test this hypothesis, we performed studies of traumatic death as a function of insurance status among adult, non-burn patients presenting to a trauma center ED. For comparison, we did parallel analysis on the same patient population in the NTDB database.

## METHODS

### Study Populations

The primary patient population was composed of trauma victims presenting to a Level 1 trauma center in a rural state (2003–2010) who were over 17 years of age, not suffering a burn injury, and not dead on arrival. We excluded patients if insurance status was missing. The final trauma center population was 13,680 patients. The initial NTDB population represented all trauma patients from 2002 through 2008. Patients were excluded if they were less than 18 years of age, dead on arrival at the ED, suffering a burn trauma, had missing or inconsistent survival status at ED or hospital discharge, or were missing insurance status. The final NTDB analysis sample was 380,182 trauma patients. Our institutional review board approved this study.

### Variables

Demographics included age, sex, and ethnicity. We categorized insurance status as commercial (managed care, commercial insurance, workers compensation), Medicare, Medicaid, and uninsured. Although patients covered by workers compensation may be otherwise uninsured, for the treatment received following their traumatic injury they had insurance coverage and were combined with the commercial insurance group as previously done. 1, 26, 32

Injury intention was coded using the Centers for Disease Control and Injury Prevention Matrix of E-code groupings (http://www.cdc.gov/injury/wisqars/ecode_matrix.html) and was categorized as intentional, unintentional or unknown. We combined self-inflicted and assault-related injuries into intentional injuries. Injury severity was measured using validated scales. The Injury Severity Score (ISS) 33 ranges from 0 to 75 and the Glasgow Coma Scale (GCS) from 3–15. 34 Higher ISS and lower GCS scores indicate greater injury severity. All reported GCS scores were at the time of ED admission.

To determine rurality, we assigned patient resident and injury zip codes the zip code approximation for the Rural Urban Commuting Area coding system. 35 Rurality was categorized as urban, large rural, small rural, and isolated rural.

### Data Analysis

We conducted analysis using SAS^®^ software, Version 9.3 of the SAS System for Microsoft, SAS Institute Inc., Cary, NC, USA. Frequencies of demographic characteristics by insurance status were calculated. No bivariate statistical tests are reported (e.g., chi-square test for proportions), as the large sample sizes resulted in statistically significant results on all variables.

The primary outcome measure was death following a traumatic injury, excluding those who died before ED arrival. For NTDB data, patients with an ED discharge disposition of “Died” and a hospital discharge disposition of “NA” or a hospital discharge of “Expired” and trauma center patients with an ED discharge disposition of “Died” and hospital discharge disposition of missing or of “Died” were coded as a traumatic death. For secondary outcome analysis, we created a variable for the location of death with values of (a) “Death in ED”, ED disposition of “died”; (b)“Inpatient Death”, ED disposition “not died”, hospital discharge “expired” (NTDB) or “died” (trauma center), and (c) “Alive at Discharge”, both ED and hospital dispositions of “not died” or “not expired”.

To estimate the relative odds of death following a traumatic injury, we calculated adjusted odds ratios (aOR) and 95% confidence intervals (CI). We excluded from analysis patients with missing data on any variable included in the model. Variables included in the model were based on *a priori* knowledge or on an association between the variable and mortality in an unadjusted model. Covariates for both patient populations were age, race, sex, injury intent, penetrating injury (Yes/No), ISS, GCS, rurality of residence, and insurance status. The co-morbidities of diabetes, cardiovascular disease, lung disease, and stroke were also included in the trauma center model. Obesity was not found to have an association with mortality in the unadjusted model and was not included. Given that the risk of mortality was likely to differ by hospital, the odds ratios using NTDB data were determined using hierarchical multivariable logistic regression analysis, controlling for correlation within a hospital.

We chose the patient’s residence zip code for the primary logistic regression model because of the following: although 61% of patient injury zip codes (8376 of 13680) were missing, among those with both zip codes (n=6191), over 63% (n=3912) had an identical injury and resident zip code. Furthermore, among the 37% (n=2,279) of patients with different injury and home zip codes, the majority (98%, 2,210 of 2,279) were state citizens injured in another area of the state.

To examine more directly whether distance from the injury zip code to the treating trauma facility affected risk of traumatic death, we then performed a logistic regression using only patients with injury zip codes. No patient zip codes were available in the NTDB, so this variable was not included in the model.

## RESULTS

### Demographics

Approximately 9% and 28% of adult, non-burn trauma center and NTDB patients were uninsured, respectively (Table 1a and 1b). The mean age of uninsured trauma center and NTDB patients was similar. As expected, Medicare patients were significantly older on average than other groups. Conversely, the mean ages for Medicaid and uninsured patients within each population were similar and several years younger than that of patients with commercial insurance. Overall, NTDB patients were more racially diverse than trauma center patients. In addition, for both trauma center and NTDB patients, there was a higher proportion of males and a lower proportion of Whites among the uninsured, as compared to the commercially insured. Among uninsured trauma center patients, 57% lived in urban, 19% in large rural, 12% in small rural and 12% in isolated rural zip codes.

### Injury Characteristics

The highest proportions of injuries for both patient populations were unintentional, regardless of insurance status (Table 2). However, uninsured and Medicaid patients from the trauma center and NTDB populations had higher proportions of intentional and of penetrating injuries, as compared to patients with commercial insurance or Medicare. In addition, when we compared uninsured patients from the trauma center with those from the NTDB, the latter had a higher proportion of intentional injuries (20% vs. 30%) and penetrating injuries (10% vs. 24%). In contrast, injury severity, as indicated by ISS and GCS scores, did not show clinically significant differences by insurance status.

### Mortality

Overall, the proportion of patients who died from traumatic injuries was similar for the trauma center (4.3%) and NTDB (4.8%). See Table 3. For both patient populations, the highest mortality rate was among Medicare patients followed by uninsured patients. Additionally, both of these mortality rates were higher than those for patients with commercial insurance or Medicaid. Among trauma center patients, a higher proportion of uninsured patients died in the ED, as compared to patients with insurance. A higher proportion of Medicare patients died after hospital admission. We saw a similar pattern among NTDB patients.

### Adjusted Odds Ratios for Traumatic Death

The relative odds of death from traumatic injury increased with age for both trauma center and NTDB patient populations (Table 4). As compared to White patients in the NTDB, Black patients were 19% more likely to die from traumatic injury (95% CI [1.03–1.38]). Among the NTDB patient population, males were 33% more likely than females to die from traumatic injury (95% CI [1.24–1.42]). Conversely, there were no differences by race or by sex for trauma center patients.

Penetrating injury was over three-fold more likely than non-penetrating injury to result in death for trauma center patients (95% CI [1.58–6.64]) and for NTDB patients (95% CI [3.36–4.29]). Increasing ISS and decreasing GCS were also associated with a higher risk of death.

With respect to co-morbidities, diabetes, cardiovascular disease, and stroke were all associated with increased odds of traumatic death among trauma center patients. Lung disease appeared to be associated with increased risk in the unadjusted model, but was not found to be so in the adjusted model. Finally, obesity was not associated in either the unadjusted or adjusted model (data not shown). None of these co-morbidities were individually associated with an increased risk of death for NTDB patients and were not included in the model.

Uninsured trauma center patients were almost five times more likely to die from traumatic injury (95% CI [2.93–8.18]) relative to patients with commercial insurance. For the NTDB, there was a 75% higher odds of traumatic death (95% CI [1.47–2.09]) among uninsured patients versus those commercially insured.

For trauma center and NTDB Medicare patients, the relative risk of death was 61% (95% CI [1.12–2.30]) and 35% (95% CI [1.22–1.51]) higher, respectively, as compared to patients with commercial insurance. There were no differences in the risk of traumatic death between patients commercially insured and those with Medicaid. Similarly, no differential risk was found among trauma center patients by rurality of patient residence.

In a sub-analysis of trauma center patients with an injury zip code (Model N=6184), there were no rurality-based differences in risk of traumatic death, after controlling for all covariates found in Table 4 (data not shown). However, the relationship between traumatic death and insurance status persisted in this model, with an almost four-fold increase in traumatic death among the uninsured (95% CI [1.96–7.82]), as compared to patients with commercial insurance.

## DISCUSSION

We observed similar demographic differences between uninsured and insured patients for trauma center and NTDB patients. The uninsured were younger than those with commercial insurance and more likely to be male. They were also more likely to be non-White. These data are consistent with 2011 U.S. census data showing that people ages 19–34 represented the highest percentage (38%) of uninsured. 2 They are also consistent with previous research on traumatic injury among the uninsured that showed disproportionate representation by the young and by males. 32 Non-Whites were also over-represented among trauma center patients relative to their percentage of the state’s population.

Trauma center patients who were uninsured or on Medicaid had higher proportions of intentional and penetrating injuries than commercially insured patients. This suggests a similar association in our state between economic insecurity and increased prevalence of intentional injury, as previously observed. 36, 37 Lack of information on socioeconomic class in the trauma center trauma registry did not allow us to test this hypothesis. Although the intent (intentional vs. unintentional) and type (penetrating *vs*. non-penetrating) of injury were different for uninsured and Medicaid patients versus commercially insured and Medicare patients, there were no clinically relevant differences in injury severity by insurance status.

Increased mortality from traumatic injury among the uninsured has been previously observed for patient populations from largely urban areas where providing emergency medical services can be challenging. Additionally, Haider et al. showed that uninsured racial minorities and penetrating trauma victims clustered at medical centers with higher mortality rates. 38 We hypothesized that an increased risk of traumatic death might not be observed for our population from a rural setting with many fewer cases of penetrating trauma. Furthermore, our state has an organized, inclusive trauma system where all hospitals are categorized (Level I–IV) based on hospital resources and capabilities. Trauma patients receive care based on clearly defined, standardized out-of-hospital and in-hospital triage criteria.

Contrary to our expectations, we found a dramatically increased adjusted risk of traumatic death among uninsured patients in our state relative to commercially insured patients. These data verify that the increased risk among the uninsured is not just a phenomenon of urban communities or of communities with more loosely organized trauma systems.

Similar to the results of Rosen et al., 1 we found that patients with Medicaid coverage were not at significantly increased risk of death following traumatic injury when compared to those with commercial insurance. Also like Rosen et al., uninsured trauma center patients and those with Medicaid were similar demographically, with a lower mean age than those commercially insured. This suggests that among younger populations having any type of insurance coverage may reduce mortality risk.

### Potential Basis for Increased Risk

Previous hypotheses to explain insurance-dependent differences in risk of death include treatment delay and differential care. 1 In 1986, the Emergency Medical Treatment and Active Labor Act (EMTALA) was enacted requiring emergency care be provided regardless of the ability to pay. This landmark legislation afforded patients of all backgrounds and circumstances the right to receive a medical screening examination and initial stabilization care for their illness, injury or labor. Despite the proven benefits of this strong anti-dumping law, there continue to be episodes where patients are inappropriately triaged, transferred to other facilities, and/or receive worse care, based on ability to pay. 16 Stronger enforcement is clearly needed to reduce these violations of patient rights.

Because of the state’s trauma system, it is unlikely that EMTALA violations account for the increased mortality risk observed for uninsured trauma center patients. Provision of emergency medical services (EMS) care and triage of trauma patients in our state follow specific guidelines that are independent of insurance status, and all patients in our data set were cared for at a single trauma center. In fact, neither EMS nor ED providers are generally aware of a patient’s insurance status at the time of treatment.

Health literacy has also been postulated as a contributor to mortality differences between commercially insured and uninsured patients. 1 Income inequality is the major contributor to differences in overall adult literacy, 39 and may be a determinant of health literacy. However, our state has fewer disparities in educational quality than many states. Moreover, income inequality and reduced health literacy might be expected to impact the mortality rate among Medicaid patients as well as the uninsured. We observed no differences in mortality between trauma center Medicaid patients and those commercially insured.

Does insurance status drive decision-making about seeking emergency care; i.e. are uninsured individuals or their families less likely or slower to dial 911? If so, that could contribute to delayed care. Studies would be needed to test this hypothesis.

We speculate that the uninsured are not a homogenous group but are rather a number of groups that may have overlapping but also unique characteristics. These groups would include healthy individuals who choose not to carry insurance because of costs or other factors, less healthy individuals who would like insurance but find it unaffordable, and individuals unable to get or have lost insurance because of pre-existing conditions.

Healthy individuals who choose not to carry insurance or who are not able to afford insurance may be younger than insured populations. Younger individuals, particularly young men, may exhibit more risk-taking behaviors that contribute to the likelihood of traumatic injury. 40 However, once injured, it is not clear why this population would be at greater risk of death, especially as they are demographically similar to Medicaid patients who are not at increased risk and we saw no insurance-dependent differences in injury severity.

Individuals with health problems may include those unable to afford insurance and those denied coverage because of pre-existing conditions. Although we controlled for several co-morbidities in our analysis, uninsured trauma victims may have undiagnosed or undocumented co-morbidities because of lack of primary healthcare and/or inadequate health records. 41 In addition, there may be a higher proportion of other co-morbidities among the uninsured that were not available for inclusion in our model that contribute to the observed differences in mortality rates. Finally, we cannot rule out the possibility that immeasurable factors exist that account for the increased risk among the uninsured.

### Relevance to Public Policy

There is an ongoing inability to identify all of the factors that contribute to insurance status-dependent differences in mortality rate, as well as proven disparities in emergency care for some uninsured patients. These are extremely difficult challenges to overcome. Studies estimating the cost effectiveness of extending health insurance coverage to the uninsured support this approach. 42 Using state level data for all 50 states from 1990–2000, researchers found that a 10% increase in coverage would predict a 1.69%–1.92% decrease in mortality and that extending private health insurance to all uninsured Americans would save over 75,000 lives and more than $400 billion each year. 42

The stated goal of the Patient Protection and Affordable Care Act (ACA) of 2010 is to reduce the number of uninsured Americans. Provisions include prohibiting insurance companies from denying or cancelling coverage for pre-existing conditions, mandating insurance coverage for individuals and businesses, providing options through federally-subsidized healthcare exchanges, and expanding the number of those eligible for Medicaid coverage. 43 In addition, the ACA calls for the establishment of new trauma center programs that strengthen ED and trauma care, support emergency medicine research, and develop innovative models for emergency care systems to reduce injury morbidity and mortality.

An important metric for measuring the effectiveness of ACA implementation will be its ability to reduce the number of uninsured and this should be accompanied by reduced mortality, including mortality specifically from traumatic injury. Having data prior to ACA implementation, such as this study, will be valuable in determining the success of the ACA.

## LIMITATIONS

Data related to potential contributors and confounders for the primary outcome (i.e. mortality) were not always available or consistent in our datasets. For example, a large proportion of NTDB records were missing information on alcohol and drug use, on the time between injury and definitive care, and whether the incident occurred in a rural or urban county. Neither trauma center nor NTDB datasets had information on household income or level of education.

In addition, there is selection bias in the NTDB because hospitals contributing data are predominately urban, have different criteria for which data are reported (e.g. deaths on admission, deaths in the ED) and different criteria for designating patients as trauma patients. With respect to generalizability, the trauma center population is largely rural and demographically homogenous and this is a single center study. These characteristics may limit the generalizability of the results. However, the observation that insurance status impacts risk of traumatic death in both the urban, racially diverse NTDB population and the rural, racially homogenous trauma center population suggests that studies looking at other sample populations are likely to find similar results. In addition, distance from and time to the treating trauma center could not be calculated due to missing data. For the trauma center population, we completed a sub-analysis for patients with an injury zip code and found no effect by rurality. However, we realize that this is an imprecise estimation of distance and time to the treatment center. Lastly, including those covered by worker’s compensation in the commercially insured group as was previously done, may have introduced bias; i.e. they may not have been otherwise insured.

## CONCLUSION

In summary, contrary to our hypothesis, uninsured trauma center patients had a higher risk of traumatic death than commercially insured patients. In contrast, Medicaid patients who were demographically similar to the uninsured and had similar types of injury were not at higher risk. Emergency medical services for trauma patients in the state and trauma care at the trauma center occur prior to knowledge of insurance status, and thus, these factors are unlikely to account for the differences. The inability to identify the basis for differences in mortality rates provides strong justification for insuring all citizens. Of note, our studies will also provide a baseline for determining the impact of the Affordable Care Act on the risk of traumatic death in our state.

## Figures and Tables

**Table 1a:** Demographics of adult, non-burn trauma patients from a Level 1 trauma center in a rural state (2003–2010) and the National Trauma Databank (NTDB) (2002–2008) by type of insurance.

	All	Commercial^1^	Medicare	Medicaid^2^	Uninsured
	
	N	n (row %)^3^	n (row %)^3^	n (row %)^3^	n (row %)^3^
Trauma center	13,680	6,996 (51%)	3,236 (24%)	2,206 (16%)	1,242 (9%)
NTDB	380,182	155,517 (41%)	89,985 (24%)	30,129 (8%)	104,551 (28%)
Age in years: Mean (SD)					
Trauma center	48 (22)	42 (17)	75 (14)	37 (13)	34 (13)
NTDB	46 (20)	43 (17)	69 (17)	39 (15)	36 (13)

1 All commercial insurance including workman’s compensation.

2 Includes state-based, income-based insurance programs.

3 Row totals may not equal study population totals due to missing values.

**Table 1b:** Demographics of adult, non-burn trauma patients from a Level 1 trauma center in a rural state (2003–2010) and the National Trauma Databank (NTDB) (2002–2008) by type of insurance.

	All	Commercial^1^	Medicare	Medicaid^2^	Uninsured
	
	n (col %)^4^	n (col %)^4^	n (col %)^4^	n (col %)^4^	n (col %)^4^
Sex					
Trauma center					
Male	8,765 (64%)	4,657 (67%)	1,536 (48%)	1,559 (71%)	1,013 (82%)
Female	4,915 (36%)	2,339 (33%)	1,700 (52%)	647 (29%)	229 (18%)
NTDB					
Male	248,094 (65%)	104,404 (67%)	42,132 (47%)	18,692 (62%)	82,866 (79%)
Female	131,818 (35%)	51,063 (33%)	47,826 (53%)	11,363 (38%)	21,566 (21%)
Race/ethnicity					
Trauma center					
White	11,758 (91%)	6,020 (92%)	3,085 (97%)	1,816 (96%)	837 (74%)
Black	437 (3.4%)	145 (2.2%)	34 (1.1%)	149 (7.1%)	109 (9%)
Hispanic	425 (3.3%)	184 (2.8%)	18 (0.6%)	88 (4.2%)	135 (12%)
Other	328 (2.5%)	180 (2.8%)	45 (1.4%)	48 (2.3%)	55 (5%)
NTDB					
White	244,502 (67%)	109,065 (73%)	72,057 (83%)	15,220 (54%)	48,160 (48%)
Black	59,947 (17%)	16,786 (11%)	8,484 (10%)	7,733 (27%)	26,944 (27%)
Hispanic	27,719 (7.6%)	9,387 (6%)	1,803 (2%)	2,505 (9%)	14,024 (14%)
Other	32,282 (9%)	13,565 (9%)	4,401 (5%)	2,979 (11%)	11,337 (11%)
Ruralty^5^					
Urban	5,560 (50%)	3,036 (52%)	1,070 (42%)	861 (48%)	593 (57%)
Large rural	2,058 (18%)	909 (16%)	568 (22%)	385 (21%)	196 (19%)
Small rural	1,687(15%)	831 (14%)	433 (17%)	295 (16%)	128 (12%)
Isolated rural	1,918 (17%)	1,050 (18%)	466 (18%)	272 (15%)	130 (12%)

1 All commercial insurance including workman’s compensation.

2 Includes state-based, income-based insurance programs.

4 Column totals may not equal study population totals due to missing values.

5 Based on 2006 Rural Urban Commuting Area (RUCA) codes for the residential zip code of the patient.

**Table 2:** Injury characteristics for adult, non-burn trauma patients from a Level 1 trauma center (2003–2010, N=13,680) and the National Trauma Databank (NTDB) (2002–2008, N=380,182) by type of insurance.

	All	Commercial^1^	Medicare	Medicaid^2^	Uninsured
	
	n (col %)^3^	n (col %)^3^	n (col %)^3^	n (col %)^3^	n (col %)^3^
Injury intent					
Trauma center					
Intentional	1,037 (8%)	293 (4%)	72 (2%)	423 (20%)	249 (20%)
Unintentional	12,596 (92%)	6,689 (96%)	3,158 (98%)	1,768 (80%)	981 (80%)
Unknown	29 (0.2%)	12 (0.2%)	4 (0.1)	8 (0.4%)	5 (0.4%)
NTDB					
Intentional	53,848 (14%)	9,658 (6%)	5,119 (6%)	7,771 (26%)	31,300 (30%)
Unintentional	324,634 (85%)	145,516 (94%)	84,648 (94%)	22,144 (73%)	72,326 (69%)
Unknown	1,698 (1%)	343 (0.2%)	217 (0.2%)	214 (1%)	924 (1%)
Penetrating injury					
Trauma center					
Yes	756 (5%)	343 (5%)	74 (2%)	219 (10%)	120 (10%)
No	12,924 (95%)	6,653 (95%)	3,162 (98%)	1,987 (90%)	1,122 (90%)
NTDB					
Yes	43,468 (11%)	8,790 (6%)	3,806 (4%)	5,677 (19%)	25,195 (24%)
No	336,714 (89%)	146,727 (94%)	86,179 (96%)	24,452 (81%)	79,356 (76%)
ISS^4^ mean (SD)					
Trauma center	12 (10)	11 (11)	13 (8.4)	11 (10)	8.6 (9.3)
NTDB	11 (10)	11 (10)	11 (8.7)	11 (11)	10 (11)
GCS^5^ mean (SD)					
Trauma center	14 (3.1)	14 (3.1)	13 (8.4)	11 (10)	14 (3.1)
NTDB	13 (4.4)	13 (4.2)	13 (4.7)	13 (4.8)	13 (4.4)

1 All commercial insurance including workman’s compensation

2 Includes state based insurance such as State papers and Iowa Cares

3 Column subtotals may not equal column total due to missing values.

4 The Injury Severity Score (ISS) is an anatomically based scoring system to provide an overall severity score for patients with multiple injuries.

5 The Glasgow Coma Scale (GCS) is a neurological scale that assesses an individual’s level of consciousness recorded at time of emergency department admission.

**Table 3:** Mortality for adult, non-burn trauma patients from a Level 1 trauma center (2003–2010, N=13,680) and the National Trauma Databank (NTDB) (2002–2008, N=380,182) by type of insurance.

	All	Commercial^1^	Medicare	Medicaid^2^	Uninsured
	
	n (col %)^3^	n (col %)^3^	n (col %)^3^	n (col %)^3^	n (col %)
Death					
Trauma center					
Yes	589 (4.3%)	200 (3%)	305 (9%)	36 (2%)	48 (4%)
No	13,091 (95.7%)	6,796 (97%)	2,931 (91%)	2,170 (98%)	1,194 (96%)
NTDB					
Yes	18,142 (4.8%)	5,264 (3%)	6,219 (7%)	1,122 (4%)	5,537 (5%)
No	362,032 (95.2%)	150,253 (97%)	82,766 (93%)	29,006 (96%)	99,007 (95%)
Death by location					
Trauma center					
Death in ED	71 (0.5%)	26 (0.4%)	28 (1%)	1 (0.1%)	16 (1.3%)
Inpatient death	518 (3.8%)	174 (2.5%)	277 (9%)	35 (1.6%)	32 (2.6%)
Alive at discharge	13,091 (95.7%)	6,796 (97.1%)	2,931 (90%)	2,170 (98.3%)	1,194 (96.1%)
NTDB					
Death in ED	2,988 (0.8%)	823 (0.5%)	454 (0.5%)	91 (0.3%)	1,620 (1.6%)
Inpatient death	15,154 (4%)	4,441 (2.9%)	5,765 (6.4%)	1,031 (3.4%)	3,917 (3.7%)
Alive at discharge	362,032 (95.2%)	150,253 (96.6%)	82,766 (93.1%)	29,006 (96.3%)	99,007 (94.7%)

*ED*, emergency department

1 All commercial insurance including workman’s compensation.

2 Includes state based insurance such as State Papers and Iowa Cares.

3 Column subtotals may not equal column total due to missing values.

**Table 4:** Adjusted odds ratios of traumatic death for trauma center (2003–2010, Model N=13,644) and for NTDB (2002–2008, Model N=378,484) patient populations.

	Trauma center		NTDB	
	
	aOR^1^	95% CI	aOR^2^	95% CI
Age				
Continuous	1.05	1.04–1.06	1.04	1.03–1.04
Race				
White	1.0 (ref)	1.0 (ref)	1.0 (ref)	
Non-White^3^	0.71	0.40–1.25	NA	NA
Black	Combined		1.19	1.03–1.38
Hispanic			1.07	0.90–1.19
Other			0.96	0.77–1.19
Sex				
Male	1.23	0.95–1.59	1.33	1.24–1.42
Female	1.0 (ref)		1.0 (ref)	
Injury intent				
Intentional	0.96	0.48–1.89	Not included	
Unintentional	1.0 (ref)			
Penetrating injury^4^				
Yes	3.24	1.58–6.64	3.79	3.36–4.29
ISS				
Continuous	1.08	1.07–1.09	1.10	1.09–1.11
GCS				
Continuous	0.74	0.72–0.77	0.85	0.83–0.87
Diabetes				
Yes	1.47	1.07–2.04	Not included	
Cardiovascular disease^4^				
Yes	1.83	1.32–2.52	Not included	
Lung disease^4^				
Yes	1.34	0.94–1.91	Not included	
Stroke				
Yes	2.89	1.58–5.30	Not included	
Insurance status				
Commercial	1.0 (ref)		1.0 (ref)	
Medicare	1.61	1.12–2.30	1.35	1.21–1.51
Medicaid	0.69	0.42–1.12	0.90	0.69–1.16
Uninsured	4.90	2.93–8.18	1.75	1.47–2.08
Rurality^5^				
Urban	1.0 (ref)		Zip codes not Available	
Large rural	0.84	0.62–1.15		
Small rural	1.03	0.73–1.46		
Isolated rural	0.89	0.63–1.26		

*NTDB*, National Trauma Data Bank; *ISS*, Injury severity score; *GCS*, Glasgow Coma Scale; *aOR*, adjusted odds ratios; *CI*, confidence interval

1 Adjusted Odds Ratios (aOR) and 95% Confidence Intervals (95% CI) were determined using logistic regression, controlling for all variables in the column. Patients were excluded if they had missing values for one or more variables.

2 aOR was determined using hierarchical logistic regression analysis to control for correlation within hospitals.

3 Due to the small number of non-Whites, other races were combined to allow for comparison.

4 Variable reference is No.

5 Rurality results based on residential zip code. No differences seen if model run with only patients having documented injury zip code.

## References

[1] Rosen H, Saleh F, Lipsitz S (2009). Downwardly mobile: the accidental cost of being uninsured. Arch Surg.

[2] DeNavas-Walt C, Proctor B, Smith J (2012). Â Income, Poverty, and Health Insurance Coverage in the United States: 2011 Current Population Reports.

[3] Franks P, Clancy CM, Gold MR (1993). Health insurance and mortality. Evidence from a national cohort. JAMA.

[4] Sorlie PD, Johnson NJ, Backlund E (1994). Mortality in the uninsured compared with that in persons with public and private health insurance. Arch Intern Med.

[5] Wilper AP, Woolhandler S, Lasser KE (2009). Health insurance and mortality in US adults. Am J Public Health.

[6] Chen AY, Schrag NM, Halpern M (2007). Health insurance and stage at diagnosis of laryngeal cancer: does insurance type predict stage at diagnosis?. Arch Otolaryngol Head Neck Surg.

[7] Chen AY, Schrag NM, Halpern MT (2007). The impact of health insurance status on stage at diagnosis of oropharyngeal cancer. Cancer.

[8] Fedeli U, Fedewa SA, Ward EM (2011). Treatment of muscle invasive bladder cancer: evidence from the National Cancer Database, 2003 to 2007. J Urol.

[9] Fedewa SA, Lerro C, Chase D (2011). Insurance status and racial differences in uterine cancer survival: a study of patients in the National Cancer Database. Gynecol Oncol.

[10] Robbins AS, Chen AY, Stewart AK (2010). Insurance status and survival disparities among nonelderly rectal cancer patients in the National Cancer Data Base. Cancer.

[11] Robbins AS, Pavluck AL, Fedewa SA (2009). Insurance status, comorbidity level, and survival among colorectal cancer patients age 18 to 64 years in the National Cancer Data Base from 2003 to 2005. J Clin Oncol.

[12] Ward E, Halpern M, Schrag N (2008). Association of insurance with cancer care utilization and outcomes. CA Cancer J Clin.

[13] Kapoor JR, Kapoor R, Hellkamp AS (2011). Payment source, quality of care, and outcomes in patients hospitalized with heart failure. J Am Coll Cardiol.

[14] Lapar DJ, Bhamidipati CM, Walters DM (2011). Primary payer status affects outcomes for cardiac valve operations. J Am Coll Surg.

[15] Mansi IA, Shi R, Altenburg R (2011). Effect of health insurance coverage on outcome for heart failure in high-risk patients. J La State Med Soc.

[16] Delgado MK, Yokell MA, Staudenmayer KL (2014). Factors Associated With the Disposition of Severely Injured Patients Initially Seen at Non-Trauma Center Emergency Departments: Disparities by Insurance Status. JAMA Surg.

[17] Alban RF, Berry C, Ley E (2010). Does health care insurance affect outcomes after traumatic brain injury? Analysis of the National Trauma Databank. Am Surg.

[18] Downing SR, Oyetunji TA, Greene WR (2011). The impact of insurance status on actuarial survival in hospitalized trauma patients: when do they die?. J Trauma.

[19] Falor A, Kim D, Bricker S (2014). Insurance status predicts survival for trauma patients undergoing urgent intervention. J Surg Res.

[20] Greene WR, Oyetunji TA, Bowers U (2010). Insurance status is a potent predictor of outcomes in both blunt and penetrating trauma. Am J Surg.

[21] Haider AH, Chang DC, Efron DT (2008). Race and insurance status as risk factors for trauma mortality. Arch Surg.

[22] Haider AH, Ong’uti S, Efron DT (2012). Association between hospitals caring for a disproportionately high percentage of minority trauma patients and increased mortality: a nationwide analysis of 434 hospitals. Arch Surg.

[23] Hakmeh W, Barker J, Szpunar SM (2010). Effect of race and insurance on outcome of pediatric trauma. Acad Emerg Med.

[24] Harris AR, Fisher GA, Thomas SH (2012). Homicide as a medical outcome: racial disparity in deaths from assault in US Level I and II trauma centers. J Trauma Acute Care Surg.

[25] Maybury RS, Bolorunduro OB, Villegas C (2010). Pedestrians struck by motor vehicles further worsen race- and insurance-based disparities in trauma outcomes: the case for inner-city pedestrian injury prevention programs. Surgery.

[26] Dozier KC, Miranda MA, Kwan RO (2010). Insurance coverage is associated with mortality after gunshot trauma. J Am Coll Surg.

[27] Rangel EL, Burd RS, Falcone RA (2010). Socioeconomic disparities in infant mortality after nonaccidental trauma: a multicenter study. J Trauma.

[28] Vettukattil AS, Haider AH, Haut ER (2011). Do trauma safety-net hospitals deliver truly safe trauma care? A multilevel analysis of the national trauma data bank. J Trauma.

[29] Tepas JJ, Pracht EE, Orban BL (2011). Insurance status, not race, is a determinant of outcomes from vehicular injury. J Am Coll Surg.

[30] (2010). How many people reside in urban or rural areas for the 2010 Census?.

[31] Barker AR, Londeree JK, McBride TD (2013). The Uninsured: An Analysis by Income and Geography.

[32] Salim A, Ottochian M, DuBose J (2010). Does insurance status matter at a public, level I trauma center?. J Trauma.

[33] Baker SP, O’Neill B, Haddon W (1974). The injury severity score: a method for describing patients with multiple injuries and evaluating emergency care. J Trauma.

[34] Teasdale G, Jennett B (1974). Assessment of coma and impaired consciousness. A practical scale. Lancet.

[b35-wjem-16-632] 35Urban Influence Codes: United States Department of Agriculture, Economic Research Service. 2013

[36] Chang SS, Stuckler D, Yip P (2013). Impact of 2008 global economic crisis on suicide: time trend study in 54 countries. BMJ.

[37] Laflamme L, Burrows S, Hasselberg M (2009). Socioeconomic differences in injury rates: A review of findings and a discussion of potential countermeasures.

[38] Haider AH, Hashmi ZG, Zafar SN (2013). Minority trauma patients tend to cluster at trauma centers with worse-than-expected mortality: can this phenomenon help explain racial disparities in trauma outcomes?. Ann Surg.

[39] Siddiqi A, Kawachi I, Berkman L (2012). Education determines a nation’s health, but what determines educational outcomes? A cross-national comparative analysis. J Public Health Policy.

[40] (2012). General Statistics: Gender.

[41] DeVoe JE, Fryer GE, Phillips R (2003). Receipt of preventive care among adults: insurance status and usual source of care. Am J Public Health.

[42] Thornton JA, Rice JL (2008). Does extending health insurance coverage to the uninsured improve population health outcomes?. Appl Health Econ Health Policy.

[43] Manchikanti L, Hirsch JA (2012). Patient Protection and Affordable Care Act of 2010: a primer for neurointerventionalists. J Neurointerv Surg.

